# Cross-sectional Survey of Hantavirus Infection, Brazil

**DOI:** 10.3201/eid1512.090229

**Published:** 2009-12

**Authors:** Jean E. Limongi, Fabíola C. da Costa, Rogério M.C. Pinto, Renata C. de Oliveira, Camila Bragagnolo, Elba R.S. Lemos, Márcia B.C. de Paula, Adalberto A. Pajuaba Neto, Marcelo S. Ferreira

**Affiliations:** Federal University of Uberlândia, Uberlândia, Minas Gerais, Brazil (J.E Limongi, F.C. da Costa, R.M.C. Pinto, M.S. Ferreira); Department of Public Health, Uberlândia (J.E Limongi, M.B.C. de Paula, A.A. Pajuaba Neto); Oswaldo Cruz Foundation, Rio de Janeiro, Brazil (R.C. de Oliveira, C. Bragagnolo, E.R.S. Lemos)

**Keywords:** Cross-sectional survey, ELISA, hantavirus, epidemiology, risk factors, Brazil, viruses, dispatch

## Abstract

A cross-sectional serosurvey was conducted to assess the proportion of persons exposed to hantaviruses in a virus-endemic area of the state of Minas Gerais, Brazil. Findings of this study suggested the presence of >1 hantaviruses circulating in this region causing hantavirus pulmonary syndrome, mild disease, or asymptomatic infection.

In Brazil, >1,080 cases of hantavirus pulmonary syndrome (HPS) have been confirmed since 1993 (case-fatality rate 40%). More HPS cases (209) are reported in the state of Minas Gerais than in any other state in Brazil (M.L. Nunes, pers. comm.). In Minas Gerais, molecular studies identified a hantavirus called *Araraquara* virus associated with HPS cases. The wild rodent *Necromys* *lasiurus* (the hairy-tailed bolo mouse, also named *Bolomys lasiurus*) was implicated as a reservoir of this virus (*1*). Because asymptomatic infection with hantaviruses also has been detected in Minas Gerais, we conducted a cross-sectional survey to assess the proportion of persons exposed to hantaviruses and to identify associated risk factors.

## The Study

The hantavirus cross-sectional survey was carried out April through May 2006 in the municipality of Uberlândia, Minas Gerais, at an average altitude of 863 m (18º55′S,48º16′W) (Figure). A randomized and stratified (sex and age) sample was collected from the entire rural area and from the south sector of the municipality’s periurban area. The term periurban refers to a residential area on the outskirts of the city that is in close contact with the rural area. The participants answered a questionnaire that included demographic information (sex, age, place of birth, and address) and questions relating to HPS risk factors (type of dwelling, exposure to rodents at home or work, labor activity, risk activities, history of severe pneumonia, and direct contact with HPS patients). Blood samples were collected by venipuncture, centrifuged, and sent to the Laboratory of Hantaviruses and Rickettsioses at the Oswaldo Cruz Foundation, Rio de Janeiro, Brazil, for analysis. The ethics review board of the Federal University of Uberlândia approved the study.

**Figure:**
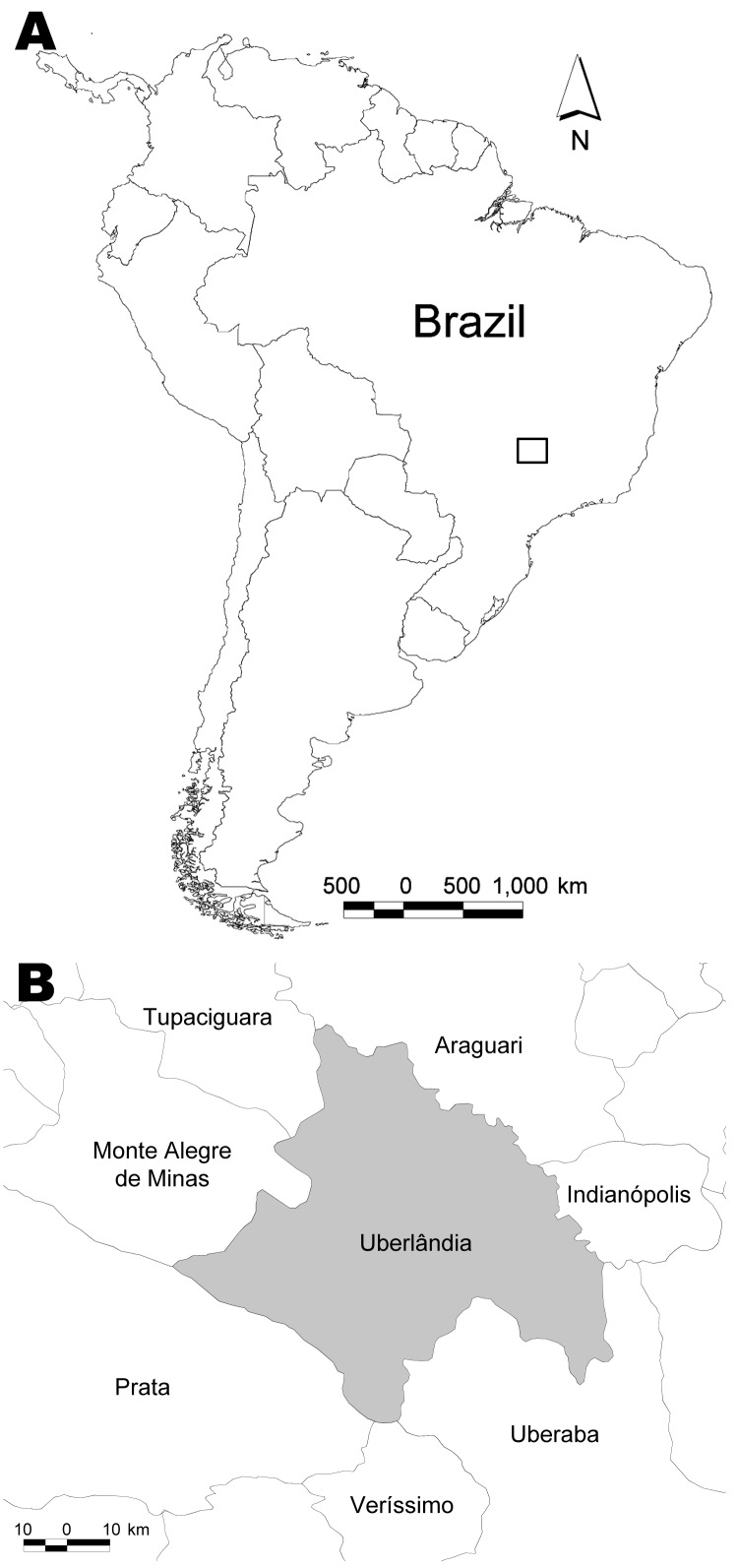
A) Location of the study area in Brazil (box). B) Detail of study area showing municipalities.

We screened serum samples by ELISA for hantavirus-specific immunoglobulin G using a recombinant antigen of the nucleocapsid protein of *Araraquara* virus, produced in *Escherichia coli* and supplied by the Virus Research Unit of the University of São Paulo, Brazil, according to the procedure previously described (*2*). All positive serum samples were retested; only those that had 3 ELISA-positive results at >1:400 dilutions were considered positive.

The Mann-Whitney U and Fisher exact/binomial tests for 2 proportions were applied for comparison among medians and proportions, respectively, using EPI INFO 3.3.2 (www.cdc.gov/epiinfo) and BIOSTAT 5.0 (www.biostat.org) software. Fisher exact test was used to estimate the odds ratio and 95% confidence intervals.

The 400 study participants comprised 200 rural and 200 periurban residents ranging in age from 12 to 76 years (mean = 41 years). Twelve (3%) samples were hantavirus antibody-positive by ELISA. The 8 rural area antibody-positive samples were from male farmers (Table 1). Presence of antibody was significantly associated with male sex, older age class, and potential risk activities (Table 1). Although all case-patients reported exposure to rodents or their excreta, this exposure was not statistically significant (Table 1). In the periurban area, the presence of antibody was associated with age but not with sex, risk activity, or exposure to rodents (Table 1). The mean age of seropositive persons from periurban and rural areas was similar (p = 1.0). The relationship between antibody and sex depended on urban vs. rural residence (p = 0.02). Three antibody-positive persons in the rural zone and 2 in the urban zone reported a history of pneumonia, albeit without complications.

**Table 1:** Relationship between independent variables and antibody to hantaviruses in the municipality of Uberlândia, Minas Gerais, Brazil, 2006

Variable	Rural			Periurban	
	No. antibody positive (no. tested)	p value*		No. antibody positive (no. tested)	p value*
Sex					
M	8 (130)	0.03		1 (84)	0.44
F	0 (70)			3 (116)	
Age, y					
12–39	0 (82)	0.01		0 (112)	0.04
>39	8 (118)			4 (88)	
Risk activity or labor†					
No activity	0 (79)	0.02		2 (92)	0.63
With activity	8 (121)			2 (108)	
Exposure to rodents					
Yes	8 (168)	0.24		3 (152)	0.67
No	0 (32)			1 (48)	

*Determined by using 2-tailed Fisher exact test. †Clearing land, farming, working in pastures or cellars, or cleaning sheds barns, or other outbuildings.

We examined data from an HPS outbreak in Uberlândia during 1998–2005. The largest number of cases occurred among periurban residents, but the highest cumulative incidence was among rural residents (Table 2). Nevertheless, rural and periurban areas did not differ significantly in either prevalence or incidence. We found higher prevalence among rural residents (Table 2).

**Table 2:** Incidence of hantavirus pulmonary syndrome and hantavirus antibody prevalence in the municipality of Uberlândia, Brazil, according to geographic area, 2006*

Variable	Area			p value†	OR (95% CI)
	Overall	Rural	Periurban		
Disease					
Cases‡	13	5	8	0.24	1.92 (0.63–5.90)
Population	71,122	17,406	53,716§		
Cumulative incidence, 1998–2005 (×10^4^)	1.83	2.87	1.50		
Infection					
Antibody positive	12	8	4	0.38	0.49 (0.14–1.65)
Sample	400	200	200		
Prevalence, % (95% CI)†	3.0 (1.3–4.7)	4.0 (1.3–6.7)	2.0 (0.1–3.9)		

*OR, odds ratio; CI, confidence interval. †Rural versus periurban. Determined by using 2-tailed Fisher exact or binomial tests for 2 proportions. ‡Limongi et al. (*3*). §Total population of the southern part of the periurban area.

## Conclusions

Overall hantavirus antibody prevalence among periurban residents was 2.0%, with a higher prevalence among women (2.6%). In previous studies, the prevalence of hantavirus antibodies was higher in men (*4*–*6*). All the positive samples in the rural area came from male farm workers. This finding is similar to a situation reported in Colombia, where all positive samples came from men engaged in rural activities (*6*). These activities involve a high risk for infection by hantaviruses (*7*).

In this study, hantavirus positivity was found only in persons >39 years of age, and the difference in the mean age of the participants in relation to positivity was significant. This fact might suggest a historic high-risk event to which the older age class, but not the younger age class, was exposed.

High hantavirus antibody prevalence has been found in studies of some human populations in Latin America (*5*,*8**,**9*). The prevalence of Araraquara virus–reactive antibodies among the volunteers in this study demonstrates that transmission is not rare, reinforcing the hypothesis of the existence of mild disease or asymptomatic infections (*10*). Two hypotheses have been proposed: clinically mild disease or inapparent infections might result from differences in the nature of exposure (e.g., low inoculum or inefficient mechanism of transmission) or genetic differences in immune response to infection, or they might indicate the circulation of >1 hantavirus genotypes of greatly reduced virulence (*10**,**11*).
